# The Weapons Identification Task: Recommendations for adequately powered research

**DOI:** 10.1371/journal.pone.0177857

**Published:** 2017-06-07

**Authors:** Andrew M. Rivers

**Affiliations:** University of California Davis, Davis, California, United States of America; University of Würzburg, GERMANY

## Abstract

This article synthesizes the extant literature on the Weapons Identification Task (WIT), a sequential priming paradigm developed to investigate the impact of racial priming on identification of stereotype-congruent and stereotype-irrelevant objects. Given recent controversy over the replicability of and statistical power required to detect priming effects, the aim of this synthesis is to systematically assess the literature in order to develop recommendations for statistical power in future research with the WIT paradigm. To develop these recommendations, the present article first quantitatively ascertains the magnitude of publication bias in the extant literature. Next, expected effect sizes and power recommendations are generated from the extant literature. Finally, a close conceptual replication of the WIT paradigm is conducted to prospectively test these recommendations. Racial priming effects are detected in this prospective test providing increased confidence in the WIT priming effect and credibility to the proposed recommendations for power.

## Introduction

Adequately powered research is important for many aspects of a cumulative science. Relative to underpowered designs, adequately powered designs yield a) greater opportunity to observe true effects (if they exist), b) lower rates of false-positives (Type I errors) in the published literature, c) more precise estimates of an effect’s magnitude, and d) greater interpretability of null-findings [1–3].

With these considerations in mind, it will be productive to establish shared power guidelines for paradigms that are commonly used in the literature [4]. The purpose of this brief review is to generate and prospectively test power recommendations specific to the Weapons Identification Task (WIT)–a commonly used sequential priming paradigm developed to investigate the influence of stereotypes on the identification of stereotype-congruent and stereotype-irrelevant objects [5]. The theory and rationale for the WIT is similar to other racial priming tasks that involve weapon identification–namely the First-Person Shooter Task [6] and the shooter computer simulation task [7]. Although they share supporting theory and rationale these measures have low correspondence to each other, which may indicate that behavioral performance on these tasks is driven by different mixtures of cognitive processes [8].

The present review has the following aims:

1. Describe the WIT paradigm and the effect of interest
2. Describe data preparation techniques as reported in the literature
3. Assess the evidential value of the published literature
4. Estimate expected effect sizes for the WIT paradigm
5. Generate recommendations for power in the WIT paradigm
6. Prospectively test recommendations for power in an independent replication

### Weapons Identification Task

The Weapons Identification Task (WIT) is a variant of sequential priming procedures adapted from cognitive psychology. Participants completing the WIT view a series of trials that consist of one of two prime faces that differ by race (Black faces or White faces) and one of two target images that differ by object-type (guns or tools). In a standard implementation of the procedure, a fixation cross precedes the presentation of a prime image for 200-ms. The prime image is directly replaced by a target image for 200-ms with no inter-stimulus interval. Finally, the target image is backward masked until a response is given. Participants render dichotomous responses to indicate having seen either a gun or a tool. Thus the WIT is a 2 (prime type: Black face versus White face) X 2 (target type: gun versus tool) within-subjects design. From here the paradigm branches to investigate the effect of racial priming on either judgment reaction times or errors in judgment (for a review see [9]).

#### Reaction time paradigm and effects

In the reaction time or ‘RT’ paradigm, participants respond as quickly as possible to identify target objects. Importantly, participant judgments in the RT variant are not constrained by a response deadline (see [5] Exp. 1). Effects reported in the literature take the form of an attenuated interaction between the two within-subjects factors (prime and target) on the reaction time to judgment. Participants correctly identify guns more quickly following Black versus White primes. In contrast, participants correctly identify tools either as quickly for both primes or in some cases more quickly following White versus Black primes (a crossover interaction).

#### Errors paradigm and effects

In the ‘Error’ paradigm, participants again respond as quickly as possible to identify target objects but must additionally register their judgments prior to a prescribed response deadline (see [5] Exp. 2). The response deadline reported in the literature ranges between 450-ms and 550-ms of target onset. Effects reported in the literature take the form of an attenuated interaction on error rates in judgment. Participants mistake tools for guns more often following Black versus White primes. Erroneous identification of guns either does not differ by prime type or, in some cases emerges as a full crossover interaction where guns are more often mistaken for tools following White versus Black primes.

### Data preparation and analysis

#### RT analyses

All reported experiments in the literature have analyzed reaction times for only correct trials [5]. Because reaction times in sequential priming paradigms generally have a positive skew, times from correct trials are log-transformed before analysis [10]. Outliers can also skew reaction time analyses [11], and researchers have adopted different upper and lower bounds for excluding reaction time outliers in the WIT paradigm (Table 1). Responses that are rendered too quickly are thought to reflect behavioral action slips and/or participant inattentiveness. Responses that are too slow can distort analyses and may also indicate participant inattentiveness. Researchers have used different strategies for handling these responses in the published literature. After log-transformation and exclusions, reaction time data aggregated at the participant level are submitted to repeated-measures ANOVA.

**Table 1:** RT paradigm data preparation and participant exclusions by experiment.

	RT Bound		
Citation	Lower	Upper	Other Exclusions
Amon & Holden, 2016	None	None	No reported exclusions
Correll, 2008	None	None	No reported exclusions
Huntsinger et al., 2009	100-ms	1000-ms	No reported exclusions
Judd et al., 2004	None	None	Exclude RT +/- 3SD outside Ss distribution
Kleiman et al., 2014	None	None	Ss with >50% errors, exclude RT +/-3SD
Kubota & Ito, 2014	None	None	Exclude RT +/- 2.5SD
Lambert et al., 2005	200-ms	None	Exclude RT +3SD
Madurski & LeBel, 2015	None	None	No reported exclusions
Payne, 2001	100-ms	1000-ms	No reported exclusions
Schlauch et al., 2009	None	None	No reported exclusions

#### Errors analyses

In comparison to reaction time analyses, relatively few exclusions have been reported in the extant Error paradigm literature. Those exclusions that have been implemented are done at the participant level to mitigate the influence of inattentive participants [12]. Researchers may also consider the possibility of excluding those participants who have low accuracy rates, who utilize a single key in responding, or who respond with a single key at rates well outside of group means. After data cleaning, error proportions are aggregated at the participant level for each of the four trial types and submitted to repeated-measures ANOVA.

## Assessing the evidential value of published literature

Before estimating an effect size for the WIT paradigm, it is important to consider and empirically test whether the reported literature likely contains evidential value and/or has been influenced by publication biases. To assess this, I first conducted a search of the literature for publications that reported data from the WIT. Studies were included in the analysis if they met two criteria. First they had to use a sequential procedure such that prime images preceded target images (i.e., SOA > 0-ms). Second, they had to use Black and White faces as prime stimuli and weapons and non-weapons as target stimuli. Searches were conducted on *PsycINFO*, *Web of Science*, and *Google Scholar* with the following keywords: *weapons task*, *weapon identification*, and *weapon AND Payne*. Additional articles were obtained via inspection of all articles that cited Payne (2001).

One technique for assessing the potential influence of publication bias is the use of the “*p*-curve” [13]. To conduct *p*-curve analyses, I aggregated *F*-statistics, their associated *p*-values, and ANOVA degrees of freedom from reported repeated-measures ANOVAs in the literature for both the RT and the Error paradigms. As an attenuated interaction is the primary prediction for both the RT and Error paradigms, the omnibus ANOVA interaction term was the statistic of interest [13]. The *p*-curve analysis plots the distribution of significant *p*-values (< .05) reported in the published literature. The shape of the distribution can then be used to infer whether there is evidential value in the published literature. A flat distribution indicates that the effect under consideration is likely “nonexistent”. In contrast, a significantly right-skewed distribution indicates that the effect under consideration likely does exist. Finally, a significantly left-skewed distribution indicates that the effect under consideration may be biased by *p*-hacking (either intentionally or unintentionally [14,15]).

### RT paradigm

**Fig 1:**
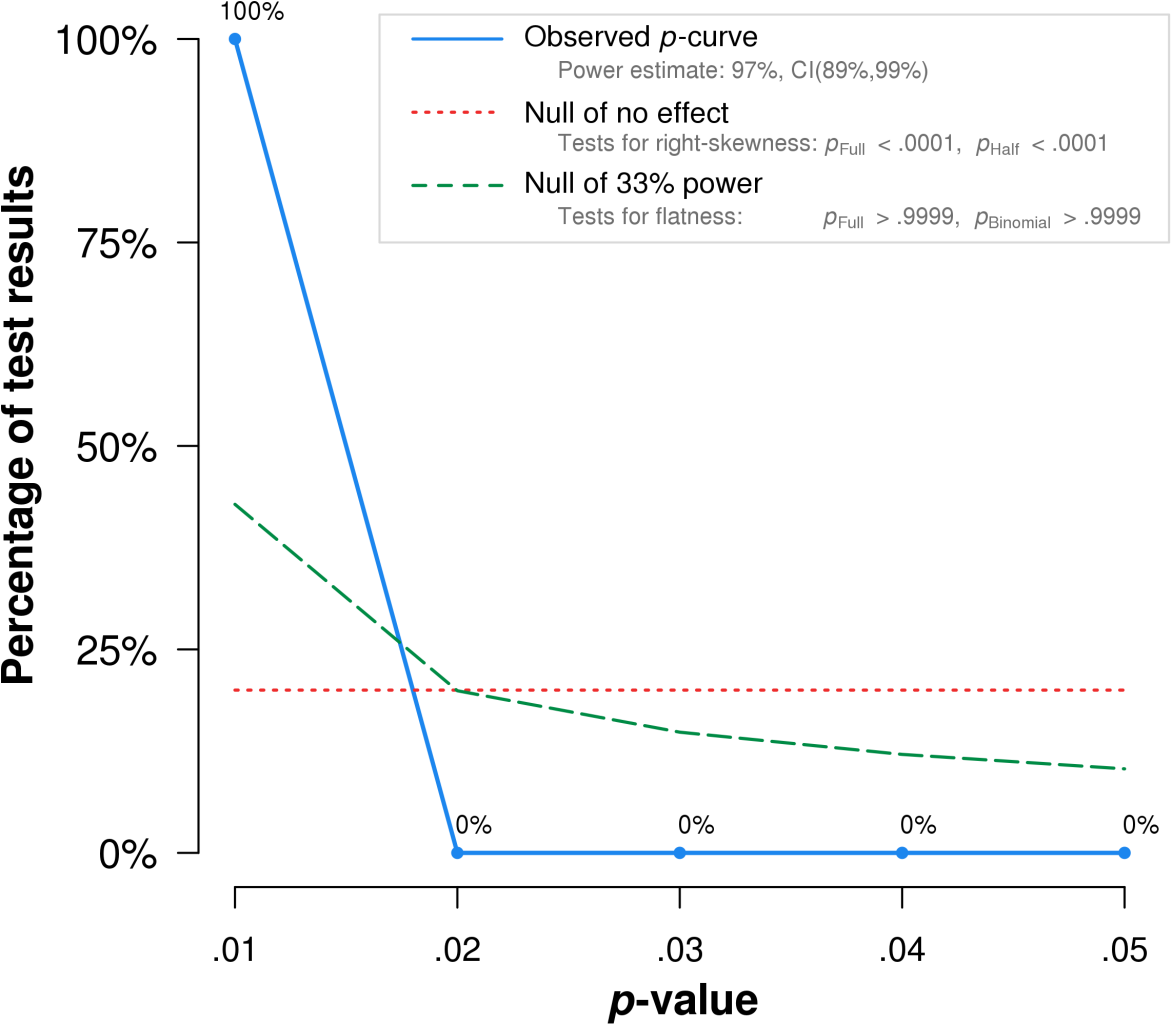
Plotted *p-*curve analyses for WIT RT paradigm.

To assess the evidential value of published experiments reporting RT effects, I aggregated 15 relevant interaction test statistics in the extant literature (Table 2). As shown in Fig 1, *p*-curve analysis (v. 4.052) for the RT paradigm indicated that the distribution had a significant right skew, *Z* = -7.67, *p* < .0001. This suggests that the WIT effect is a) likely to exist, and b) unlikely biased by extensive *p*-hacking.

**Table 2:** Reported experimental data by WIT paradigm type, number of participants, and number of task trials per participant.

Article	Exp.	RT	ERR	N	Trials
Amodio (2009) [16]	1		X	35	188
Amodio et al. (2004) [17]	1		X	34	288
Amodio et al. (2006) [18]	1		X	66	144
Amodio et al. (2008) [19]	1		X	45	288
Amon & Holden (2016) [20]	1	X		128	1100
Bartholow et al. (2012) [21]	1		X	65	384
Bradley & Kennison (2012) [22]	1		X	87	128
Camp et al. (2015) [23]*	1		X	72	80
Correll (2008) [24]	2	X		71	200
Fleming et al. (2010) [25]*	1		X	33	120
Gorovun & Payne (2006) [26]	1		X	72	128
Huesmann et al. (2012) [27]*	1		X	269	208
Huntsinger et al. (2009) [28]	1	X		82	160
Ito et al. (2015) [8]	1		X	401	384
Jones & Fazio (2010) [29]*	1–3	X	X	323	160
Judd et al. (2004) [30]	1	X		59	480
Klauer & Voss (2006) [31]	1		X	40	480
Klauer et al. (2015) [32]	5		X	156	720
Klauer et al. (2015) [32]*	6		X	48	240
Kleiman et al. (2014) [33]	2	X		44	256
Kubota & Ito (2014) [34]	1		X	71	360
Kubota & Ito (2014) [34]	2	X		166	120
Lambert et al. (2003) [35]	2		X	127	384
Lambert et al. (2005) [36]	2	X		60	384
Madurski & LeBel (2015) [37]	1	X		296	200
Payne (2001) [5]	1	X		31	192
Payne (2001) [5]	2		X	32	192
Payne (2005) [38]	1		X	69	128
Payne (2005) [38]	2		X	55	320
Payne et al. (2002) [39]	1		X	93	384
Payne et al. (2005) [40]	1		X	33	192
Payne et al. (2005) [40]	2		X	33	192
Schlauch et al. (2009) [41]	1	X	X	89	256
Stepanova et al. (2012) [42]	1		X	171	192
Stewart & Payne (2008) [43]	1		X	146	192
Stewart & Payne (2008) [43]	2		X	125	192
Todd et al. (2016a) [44]	1		X	143	288
Todd et al. (2016b) [45]	1		X	63	144
Todd et al. (2016b) [45]	2		X	125	288

* Denotes studies excluded from bias and meta-analyses.

### Error paradigm

**Fig 2:**
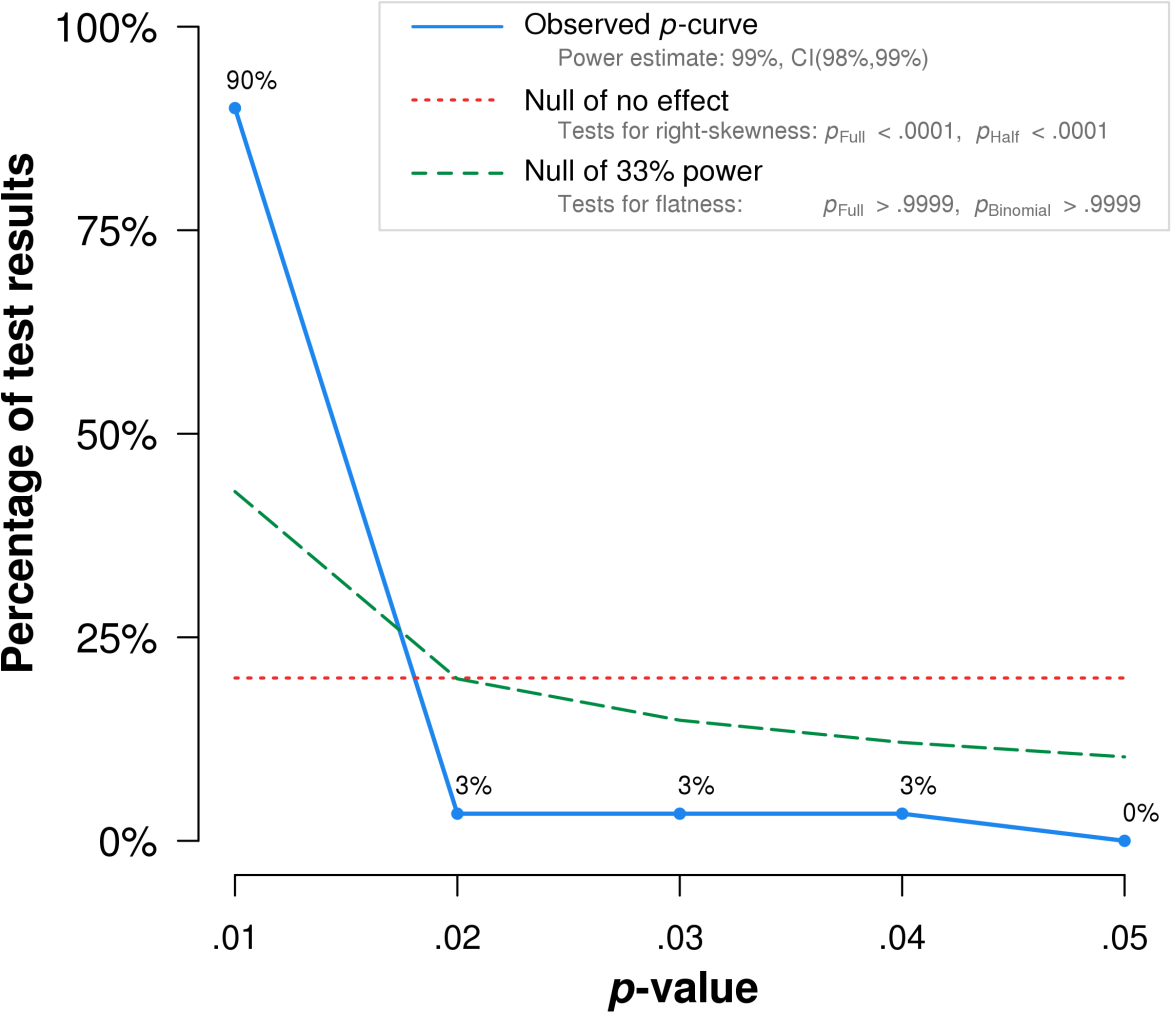
Plotted *p-*curve analyses for WIT Error paradigm.

I aggregated interaction test statistics from 33 interaction test statistics in the extant literature (Table 2). As shown in Fig 2, the results of the *p*-curve analysis for the Error paradigm indicated that the curve had a right skew, *Z* = -15.85, *p* < .0001.

## Estimating WIT effect size

Results from the *p*-curve analyses indicated that the extant literature a) likely contained evidential value, b) was not detectably biased by intense *p*-hacking, and c) appeared highly-powered to detect the effect. The results of this analysis suggest that the effect sizes reported in the literature would be informative in estimating the effect sizes. Thus, effect sizes were computed for each published experiment. Researchers must decide which studies should be included in the estimate of each type of effect. As an example, Stewart and Payne [24] implemented an intervention intended to eliminate stereotypic biases and, therefore, the effect of interest. Notably, this intervention fell short of entirely eliminating the WIT effect, but arguably should not be included in calculating an average expected effect size for close replications of WIT that do not use this intervention. Likewise, some interventions sought to determine if situational manipulations (e.g., alcohol) would increase WIT bias, and these arguably should not be included. Thus for the present effect size analyses, I report estimations that first include all available experimental data. In a subsequent analysis, report estimations that only include experiments that I subjectively considered as close replications of the paradigm (excluding those that sought to attenuate or exacerbate WIT effects). Examples of close replications can include experimental data with minor modifications (e.g., [20,32]) and those paradigms that used the WIT to document individual differences (e.g.,[8,19]).

WIT effect sizes were estimated by fitting random-effects models in the ‘metafor’ package in the R statistical computing environment [46,47]. Each model accounted for nesting of experimental data set within reported studies. Additional robustness checks indicated that other plausible nesting of the data (e.g., by corresponding author) did not substantively impact the reported estimates. Results for both the RT and Error paradigms indicated that the interaction was reliable and that heterogeneity was detectable for each of the analyses (see Tables 3 and 4). Finally, funnel plot asymmetry tests did not detect bias in the RT literature (*t*(13) = -.118, *p* = .908); nor did it detect bias in the Error literature (*t*(31) = .087, *p* = .931). This comports with conclusions from the *p-*curve analyses using a more traditional metric for assessing publication bias.

**Table 3:** Estimated effect size of the RT paradigm by inclusion criterion.

Inclusion criterion:	Pearson’s *r*	*η*^2^_partial_	Heterogeneity
All studies	.326 [.197,.455]	.106 [.039,.207]	*Q*(14) = 64.6, *p* < .001, *I*^2^ = .811
Close replications	.399 [.279,.519]	.159 [.077,.270]	*Q*(10) = 28.9, *p* = .001, *I*^2^ = .716

Note: Each estimate is bounded by 95% confidence interval.

**Table 4:** Estimated effect size of the Error paradigm by inclusion criterion.

Inclusion criterion:	Pearson’s *r*	*η*^2^_partial_	Heterogeneity
All studies	.452 [.389,.515]	.204 [.151,.266]	*Q*(32) = 68.6, *p* < .001, *I*^2^ = .562
Close replications	.477 [.411,543]	.228 [.169,.295]	*Q*(25) = 46.3, *p* = .006, *I*^2^ = .489

Note: Each estimate is bounded by 95% confidence interval.

## Power recommendations

Power estimates were calculated using G*Power v3.1 [48]. Recommendations for number of participants are shown in Tables 5 and 6. There are several notes regarding their interpretation. First, these estimates of sample size are only for observing the fully within-subjects interaction in each of the WIT paradigms. Studies investigating the impact of situational interventions very likely need to be powered at much higher N than the present recommendations. Consider that the most effective intervention to reduce the WIT effect was unsuccessful in doing so [24]. In fact, there was still an observable interaction in the Error paradigm, albeit with an attenuated effect size (*ω*^2^ = .045, *η*^2^_partial_ = .063). In contrast, interventions emphasizing quick responding have produced WIT effects that were only directionally stronger than the estimated average effect size (*ω*^2^ = .383, *η*^2^_partial_ = .398). Thus, when powering experiments to investigate bias-interventions specifically, expect effect sizes to range between *ω*^2^ = .04 and *ω*^2^ = .40. Given this range, many more participants per experimental level may be needed to investigate the impact of between-subjects interventions.

**Table 5:** Recommendations for power in number of participants for RT paradigm (1-*β* = 80% and 95%) by inclusion criterion.

	1-*β* = .80	1-*β* = .95
All studies	57 [28, 157]	97 [47, 273]
Close replications	37 [21, 78]	63 [35, 136]

Note: Parentheses indicate N required at upper and lower bounds of the estimated effect size.

**Table 6:** Recommendations for power in number of participants for Error paradigm (1-*β* = 80% and 95%) by inclusion criterion.

	1-*β* = .80	1-*β* = .95
All studies	28 [22, 40]	46 [34, 66]
Close replications	26 [20, 34]	42 [30, 58]

Note: Parentheses indicate N required at upper and lower bounds of the estimated effect size.

Scientists investigating statistical power in replication and research design have differing recommendations with respect to power in experimental work. For example, Simonsohn’s [49] small telescopes approach recommends at least 2.5x original sample sizes when attempting to replicate previous experimental work. As another example, Lakens and Evers [50] have put forward sequential analysis techniques designed to control Type I error rates while conserving scarce data collection resources. These and many more approaches are available that seek to balance the precision of parameter estimation and the allocation of researcher resources. In this vein, it is important to point out that the present power recommendations assume that meta-analytic effect size estimates are not biased by selective reporting. Although *p*-curve and funnel plot analyses did not detect systematic bias, this does not necessarily indicate that no bias is present. Thus, it remains possible that WIT effect sizes are upwardly biased in the reported literature. Researchers can and should use these recommendations as a starting point, modifying their sampling plan as needed based on available resources, desire for certainty or precision in estimation, and as additional data becomes available.

## Independent replication of Payne (2001)

To complement this analysis of the literature and recommendations for power, I conducted an independent replication of the two experiments reported in Payne [5]. In doing this, I sought to prospectively investigate the efficacy of my power recommendations for the WIT RT and Error paradigms.

### Research design

The design of this replication can be considered “close” but not “exact.” The differences between the replication and Payne [5] are as follows. First, I utilized previously validated racial prime stimuli that have not yet been investigated in the WIT literature [51]. The total set consisted of head and shoulders color photographs of 24 Black males and 24 White males. Second, I generated new target stimuli of both weapons and tools. These stimuli consisted of 5 guns and 5 tools. To reduce the possibility that participants could identify targets based on repeated presentation, I rotated each image by 90 degrees to produce 4 orientations for a total set of 40 target stimuli. Finally, I implemented a third set of neutral control prime images that consisted of the outline of a face (see [52]).

It is possible that each of these modifications might produce results that diverge from that of the original paradigm. However, any differences these modifications produce would be informative when considered on a conceptual level. If we observe WIT effects with a) new prime stimuli, b) new target stimuli, and c) a new class of prime stimuli; we can then have increased confidence in theories that propose priming race produces differences in the speed and accuracy of identifying guns and tools may be generalized beyond the simple specification implemented in the original reported study [53]. If we do not observe WIT effects with these modifications, then theory must be constrained to reflect boundary conditions of the effect (e.g., “the effect does not occur with different target stimuli”).

With the exception of the aforementioned differences, all other aspects of the procedure closely parallel those reported in Payne [5]. Participants completed the WIT protocol at individual cubicles in groups of 1–4. After providing informed consent, participants were informed that they were participating in a task investigating visual perception. After completing 18 practice trials each, participants all completed 216 critical test trials. On each trial, a visual fixation cross appeared for 500-ms. The fixation cross was replaced by a prime image presented for 200-ms. The prime was directly replaced by a target image presented for 200-ms. The target image was backward masked by a visual static image until a response was given. Participants received two self-paced breaks after each block of 72 critical trials. Finally, as a between-subjects manipulation, participants were either assigned to complete the task with a 500-ms response deadline or a 1000-ms response deadline. Whereas Payne [5] compared across two independent experiments, the between-subject manipulation of the response deadline allows for comparisons between the two conditions. For responses registered beyond the deadline, participants saw the message “Please try to respond faster!” for 2-seconds.

### Participants

Given my recommendations for power in the two paradigms I sought to collect data from at least 40 participants each for the RT and Error paradigms. The data were not analyzed prior to surpassing the desired sample size. Eighty-seven undergraduates from UC-Davis participated in exchange for partial course credit (92 percent female; 55 percent Asian, 23 percent Latino/a, 17 percent White, 2 percent Black, and 2 percent unidentified). All participants gave written consent to participate and all study procedures were approved by the University of California Davis Office of Research. A computer error resulted in uninterpretable data for 5 participants, thus the final data set consisted of 42 participants in the 1000-ms condition (RT condition) and 40 participants in the 500-ms condition (Error condition). I set *a priori* criterion to exclude participants who used a single key in responding to all trials, but no participants met this criterion. Full data are available from OSF at osf.io/9e6sa/.

### Results

#### RT analysis

The analysis plan is identical to that reported in Payne [5]. Only accurate identifications were included and reaction times less than 100-ms and greater than 1000-ms were trimmed from the analysis (4.62% of data for 1000-ms condition; 20.56% for 500-ms condition). A log transformation was applied to reduce positive skew in the resulting distribution [5]. Mean reaction times were aggregated for each trial type and subjected to mixed model ANOVA with response deadline (500-ms vs. 1000-ms) as a between-subjects factor and prime (Black vs. White) and target (gun vs. tool) as within-subjects factors.

As in Payne [5], there was a main effect of target type, *F*(1,80) = 63.467, *p* < .001, *η*^2^_partial_ = .442, 95%*CI*_difference_[.080,.133] (see Table 7). Weapons were correctly identified faster than tools. This main effect was qualified by the critical prime x target interaction, *F*(1,80) = 22.965, *p* < .001, *η*^2^_partial_ = .223. Tests of simple effects indicated that guns were identified more quickly following Black versus White primes, *t*(81) = 4.176, *p* < .001, *CI*_difference_[.034,.095]. Likewise, tools were identified more quickly following White versus Black primes, *t*(81) = 3.576, *p* = .001, *CI*_difference_[.031,.108]. This pattern of results is consistent with a crossover interaction (rather than an attenuated interaction). Finally, there was a main effect of response deadline. The 500-ms deadline produced faster identifications than the 1000-ms deadline, *F*(1,80) = 28.715, *p* < .001, *η*^2^_partial_ = .264, *CI*_difference_[.142,.309]. No other interactions approached statistical reliability.

**Table 7:** Mean log-transformed RT by trial type and by level of response deadline.

	Prime					
	Black			White		
Target	*M*	*SE*	*95% CI*	*M*	*SE*	*95% CI*
500-ms deadline						
Gun	5.444	.036	[5.372,5.517]	5.499	.033	[5.432,5.566]
Tool	5.615	.032	[5.550,5.680]	5.541	.040	[5.459,5.622]
1000-ms deadline						
Gun	5.660	.033	[5.593,5.727]	5.733	.032	[5.667,5.798]
Tool	5.836	.029	[5.777,5.894]	5.771	.032	[5.706,5.837]

#### Error analysis

The analysis plan is again identical to that reported in Payne [5]. The numbers of errors were aggregated by each prime-target combination and subjected to mixed model ANOVA. The overall rate of errors was higher in the 500-ms response deadline condition versus 1000-ms, *F*(1,80) = 56.179, *p* < .001, *η*^2^_partial_ = .413, *CI*_difference_[.146,.251] (35.91% vs. 16.04% respectively; see Table 8). There was a main effect of target type, *F*(1,80) = 24.057, *p* < .001, *η*^2^_partial_ = .231, *CI*_difference_[.046,.109]. Tools were more often misidentified than guns, which can be interpreted as a response bias in favor of guns. This effect was qualified by a target x response deadline interaction, *F*(1,80) = 13.331, *p* < .001, *η*^2^_partial_ = .143. This interaction suggests a response bias in favor of guns was exacerbated when the response deadline was shorter. Replicating Payne [5], the main effect of target was qualified by the critical prime x target interaction, *F*(1,80) = 10.392, *p* < .001, *η*^2^_partial_ = .115. Simple effects indicated that tools were more often misidentified following Black versus White primes, *t*(81) = 2.937, *p* = .004, *CI*_difference_[.019,.100], whereas guns were more often misidentified following White versus Black primes, *t*(81) = 2.519, *p* = .014, *CI*_difference_ [.008,.069]. Finally as implied by Payne [5], a three way prime x target x response deadline interaction was marginal, *F*(1,80) = 3.831, *p* = .054, *η*^2^_partial_ = .046. This higher order interaction indicates that *stereotype-congruent errors* were more frequent for the 500-ms deadline than for the 1000-ms deadline.

**Table 8:** Mean proportion of errors by trial type and by level of response deadline.

	Prime					
	Black			White		
Target	*M*	*SE*	*95% CI*	*M*	*SE*	*95% CI*
500-ms deadline						
Gun	.254	.016	[.221,.287]	.329	.020	[.288,.369]
Tool	.468	.035	[.398,.538]	.385	.032	[.320,.451]
1000-ms deadline						
Gun	.148	.023	[.102,.194]	.153	.020	[.113,.192]
Tool	.187	.026	[.135,.240]	.153	.024	[.105,.202]

#### Analysis of critical predictions

To enhance the direct comparability of the present replication with Payne [5], I analyze the critical prime x target interactions for the RT and Error paradigms separately. As described in the present literature, the RT effect should be most evident when the response deadline is longer (1000-ms) compared to when it is shorter (500-ms). In contrast, the Error effect should be most evident when the response deadline is shorter compared to when it is longer.

**Fig 3:**
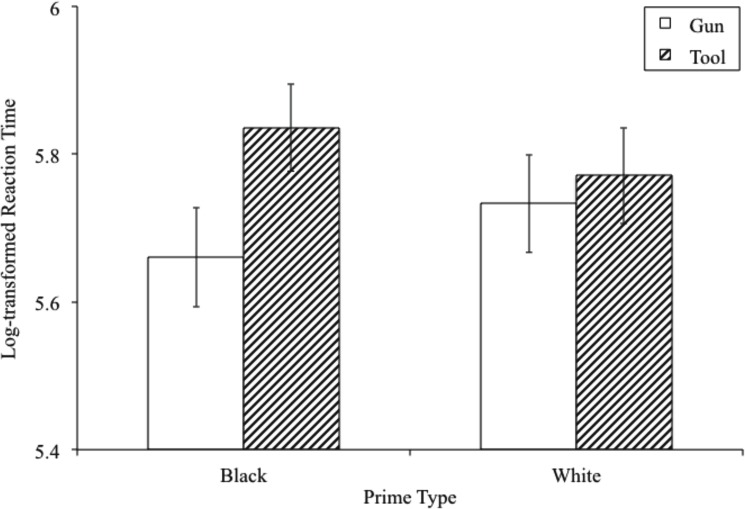
Log-transformed reaction time by prime and target at 1000-ms response deadline.

When the long response deadline was imposed, the expected prime x target interaction on reaction times was observed, *F*(1,41) = 35.395, *p* < .001, *η*^2^_partial_ = .463 (see Fig 3). Note that this effect is stronger than expected, and falls outside the confidence interval, given estimates from the meta-analytic estimate. Both simple effects were detectable. Guns were identified more quickly following Black versus White primes, *t*(41) = 4.441, *p* < .001, *CI*_difference_[.040,.106], and tools were identified more quickly following White versus Black primes, *t*(41) = 4.372, *p* < .001, *CI*_difference_[.035,.094]. As discussed above, the effect was not statistically moderated by the response deadline factor.

**Fig 4:**
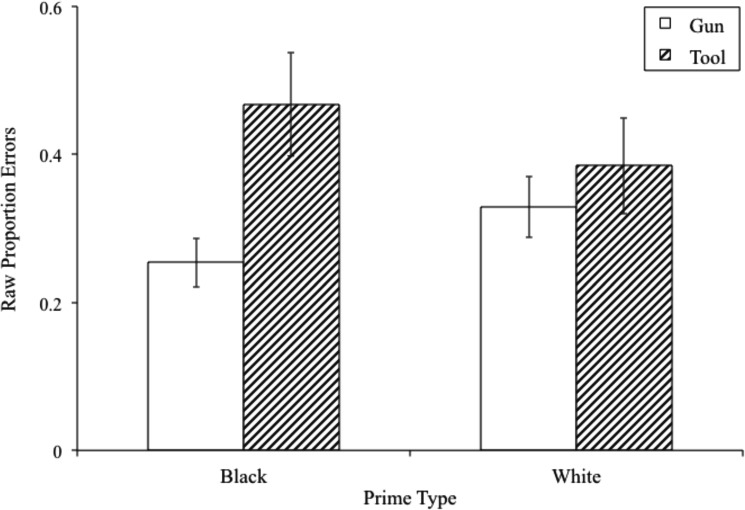
Proportion errors by prime and target at 500-ms response deadline.

When the 500-ms response deadline was imposed, the expected prime x target interaction on error rates was observed, *F*(1,39) = 7.939, *p* = .008, *η*^2^_partial_ = .169 (see Fig 4). Note that this effect falls within the confidence interval of estimated effect size from the meta-analysis. Both simple effects were detected. Tools were more often misidentified following Black versus White primes, *t*(39) = 2.232, *p* = .031, *CI*_difference_[.008,.158], and guns were more often misidentified following White versus Black primes, *t*(39) = 2.819, *p* = .008, *CI*_difference_[.021,.123]. As described above, the effect was moderated by response deadline in the predicted direction–stereotype-congruent errors were more common when the response deadline was 500-ms versus when it was 1000-ms.

## Discussion

This brief review evaluated the reported literature investigating the Weapons Identification Task, a commonly-used sequential priming task. The review indicated that 1) there are differences in implementation and analysis of data in the paradigm and that 2) the published literature investigating the WIT paradigm very likely contains evidential value despite these differences and is not substantially impacted by publication bias. Given the favorable results of the publication bias analysis, I used effects reported in the extant literature to generate estimates of effect sizes for both the RT and Error WIT paradigms. Using estimated effect sizes I then generated recommendations for adequate power in each paradigm. The appropriateness of this strategy is contingent on the assumption that publication bias has not contaminated the literature. In many cases, this assumption may prove problematic; however, the *p*-curve analyses supported this assumption in the present analysis.

Finally, I tested the efficacy of these recommendations prospectively by conducting a close independent replication of both the RT and Error paradigms. Both the RT as well as the Error interactions emerged in this prospective test, supporting the published literature and the proposed recommendations for power. Notably, the size of the RT effect was stronger than expected given the meta-analytic findings. The size of the Error effect fell within the confidence interval of the meta-analysis. These recommendations are not static and should be flexibly revised as additional data becomes available.

There are several limitations of the present work that should be noted. First, the reported meta-analytic estimates depend on the assumption that selective reporting has not biased published WIT effects. Tests of this assumption found no evidence for bias in the literature. However, there are relatively few significant interaction test statistics in the RT literature (k = 9) and therefore less power to detect bias. Simonson et al. [13] reports simulations of this case that suggest k = 10 is sensitive enough to find evidence that a set of studies lacks evidential value, especially when the literature appears to have highly powered studies (as appears to be the case with the WIT literature). Even if the reported literature is found to contain evidential value, we cannot be certain that effects in the reported literature are not upwardly biased. This possibility should be assessed as additional evidence is collected investigating WIT and as the *p*-curve technique is further probed and refined. It is also important to explicitly acknowledge the strengths and limitations of the current experimental replication. Although implementation of the WIT paradigm and corresponding data analytic techniques are relatively constrained, this does not rule out the possibility that researcher decisions can influence (even unconsciously) the interpretation of results [14]. So that others may independently evaluate the strength of the replication evidence I would like to reiterate that the sample size, task implementation (e.g., stimuli), and data analytic strategies were decided prior to data collection. Additionally the data were not examined at any intermediate point prior to the critical analyses. However, a limitation is that these plans were not preregistered on a public site. Preregistration is considered by some to be a powerful mechanism for increasing confidence in published results [54]. It is therefore appropriate for a skeptical reader to consider this when evaluating the current replication results.
